# DNA replication stress triggers rapid DNA replication fork breakage by Artemis and XPF

**DOI:** 10.1371/journal.pgen.1007541

**Published:** 2018-07-30

**Authors:** Rémy Bétous, Théo Goullet de Rugy, Alessandra Luiza Pelegrini, Sophie Queille, Jean-Pierre de Villartay, Jean-Sébastien Hoffmann

**Affiliations:** 1 CRCT, Université de Toulouse, Inserm, CNRS, UPS; Equipe labellisée Ligue Contre le Cancer, Laboratoire d’excellence Toulouse Cancer, Toulouse, France; 2 Department of Microbiology, Institute of Biomedical Sciences, University of São Paulo, São Paulo, Brazil; 3 Laboratory “Genome Dynamics in the Immune System”, INSERM UMR1163, Université Paris Descartes Sorbonne Paris Cité, Institut Imagine, Paris, France; MRC Laboratory of Molecular Biology, UNITED KINGDOM

## Abstract

DNA replication stress (DRS) leads to the accumulation of stalled DNA replication forks leaving a fraction of genomic loci incompletely replicated, a source of chromosomal rearrangements during their partition in mitosis. MUS81 is known to limit the occurrence of chromosomal instability by processing these unresolved loci during mitosis. Here, we unveil that the endonucleases ARTEMIS and XPF-ERCC1 can also induce stalled DNA replication forks cleavage through non-epistatic pathways all along S and G2 phases of the cell cycle. We also showed that both nucleases are recruited to chromatin to promote replication fork restart. Finally, we found that rapid chromosomal breakage controlled by ARTEMIS and XPF is important to prevent mitotic segregation defects. Collectively, these results reveal that Rapid Replication Fork Breakage (RRFB) mediated by ARTEMIS and XPF in response to DRS contributes to DNA replication efficiency and limit chromosomal instability.

## Author summary

DNA replication is an essential process that needs to be absolutely accurate to prevent fixation of mutations which could impair cellular essential functions and promote diseases such as cancers. During S-phase DNA replication forks encounter many obstacles that block the replicative DNA polymerases and induce fork stalling. Accumulation of stalled forks or excessive fork slowing is referred to as DNA replication stress which promote a DNA damage response elicited by ATR from the stalled forks to preserve genome stability. However, how cells deal with persistently stalled replication forks is not fully understood. It has been shown that the endonuclease MUS81-EME1 can cleave the stalled forks after 24 hours of replication stress. However normal S-phase length, is commonly of about 8 hours. Thus we asked what could happen if forks stall more transiently. We uncovered that stalled DNA replication forks can break rapidly after induction of replication stress. We show that this Rapid Replication Fork Breakage (RRFB) is achieved by two endonucleases, ARTEMIS and XPF-ERCC1, which work independently of each other to resume DNA replication from the stalled forks and to prevent mitotic segregation defects. Hence, we identified new pathways preserving genome stability during replication stress.

## Introduction

Genome stability is affected not only by exogenous aggressions such as chemical carcinogens and ionizing radiation but also by endogenously induced DNA damage generated during the process of DNA replication when the DNA replication forks are slowed down or stalled by diverse natural replication barriers, a phenomenon referred as DNA replication stress (DRS) [1]. This has been well documented during cancer progression, in which oncogene-driven cell proliferation induces a high level of DRS, resulting in the genome instability that is a hallmark of cancer cells [2]. DRS generates stalled replication forks containing large amounts of single-stranded DNA coated with the protein RPA. These activate the replication stress kinase ATR, which phosphorylates hundreds of substrates in order to stabilize and restart the stalled DNA replication forks [3]. If this replication stress response fails, DNA double-strand break (DSB)s are created at the stalled replication forks [4], a phenomenon known as replication fork breakage. Currently, the mechanisms mediating replication fork breakage are poorly understood and have only been reported to occur in response to extensive DRS where it requires MUS81 [5, 6], the catalytic subunit of a structure-specific endonuclease which forms a complex with EME1 or EME2 [7].

DSBs are usually seen as threats to genome integrity and cell viability, so it is unclear why eukaryotes have evolved mechanisms that induce DSBs during DNA replication. A recent body of evidence, however, suggests an answer to this conundrum. Two studies have found that DRS can lead to under-replicated regions that persist into late G2 and M phase of the cell cycle [8, 9]. If these regions remained unresolved in mitosis, these can lead to chromosomes segregation defects, which can result in uncontrolled chromosome breaks that are transmitted to daughter cells upon cell division, thus affecting the genomic stability of the next cell generation. Importantly, Mus81 was demonstrated to prevent these detrimental consequences of DRS [10, 11].

Initially, MUS81 dependent fork breakage has been observed after prolonged exposure to DRS (18–24 hours) and has been proposed to occur in S-phase cells [5]. However, even though MUS81 exhibits basal activity throughout the cell cycle, its main biological function seems to be restricted to mitosis to promote the resolution of under-replicated chromosomes, holliday junctions or other recombination intermediates [7] and mistargeting of MUS81 to replication factories during S-phase induces chromosome pulverization [12]. Therefore, whether stalled replication forks are actually subjected to endonucleolytic cleavage during S-phase remains unclear. Interestingly, activation of the DNA damage response and recruitment of DSB repair proteins to stalled replication forks occur as early as 2 hours after induction of DRS [13] suggesting that at least some stalled replication forks are being cleaved within this timeframe. Thus, we investigated if DNA replication forks could actually break earlier than previously reported, when S-phase is challenged by replication inhibitors.

In this study, we provide evidence that Replication forks can break quickly in S-phase upon DRS induction by an endonucleolytic mechanism independent of MUS81. We demonstrate that two nucleases ARTEMIS and XPF-ERCC1 are responsible for this Rapid-Replication Fork Breakage (RRFB) which takes place during S and G2 phases of the cell cycle. We propose that this physiologically-controlled DSB induction is important to prevent genetic instability under DRS.

## Results

### Replicative stress induces rapid replication fork breakage

**Fig 1:**
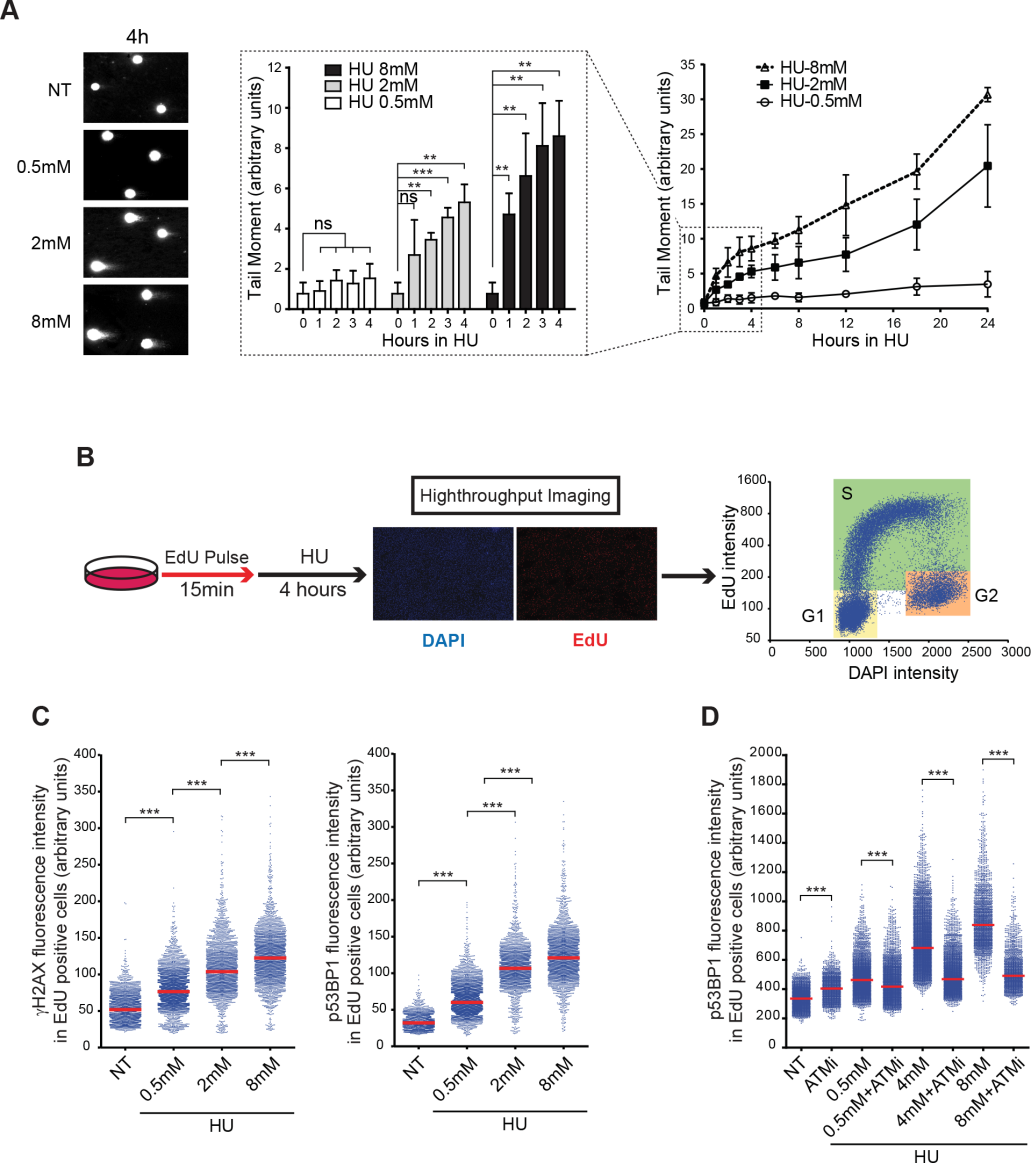
Brief replication stress results in rapid replication fork breakage. (A) Quantification by neutral comet assay of DSBs induced, in response to treatment with various doses of HU for up to 24 hours. Representative images of cells treated for 4 hours are shown. Each data point corresponds to the mean tail moment (% DNA in tail x tail length) and error bars represent standard deviations from 3 independent experiments in which at least 150 comets were scored. (B) Schematic representation of quantitative image-based cytometry (QIBC). (C) Quantification of nuclear γH2AX (left) or p53BP1 (right) fluorescence intensity by QIBC in >2500 S-phase cells treated with the indicated doses of HU for 4 hours. ***p < 0.0001 (Mann-Whitney test). (D) Quantification of nuclear p53BP1 fluorescence intensity by QIBC in > 2000 S-phase cells either untreated (NT) or pretreated for 1 hour with an ATM inhibitor (ATMi; KU55933 10μM) and then treated with the indicated concentrations of HU for 4 hours in the presence or absence of the ATM inhibitor. ***p < 0.0001 (Mann-Whitney test).

To investigate the kinetics of replication fork breakage, we used the sensitive neutral comet assay to monitor the occurrence of DSBs at various levels of replicative stress. We treated the human colon carcinoma cell line RKO with 0.5, 2 and 8 mM hydroxyurea (HU), an inhibitor of dNTP biogenesis, for 1–24 hours. This DRS induced DSBs detectable as early as 1 hour after HU treatment at the strongest dose of HU (Fig 1A). During the first four hours of DRS, the amount of DSBs increased linearly and reached a plateau before increasing again from 16–24 hours. DSBs observed during the earliest time points of the assay appeared concomitant with activation of DNA-PK, which is known to be activated by DSBs, and to the phosphorylation of one of its substrates, RPA, on Ser 4 and 8 (S1A Fig).

To determine whether the observed DSBs were induced in S-phase cells, we carried out quantitative image-based cytometry (QIBC), a high-throughput microscopy method described in Fig 1B [14]. QIBC allowed us to quantify in the individual cells of a population the amounts of several DNA damage markers and to correlate this with the stage of the cell cycle. After 4 hours of DRS induced by HU, we observed, as previously described [14], a strong, dose-dependent accumulation of γH2AX, the phosphorylated form of the histone variant H2AX, which is phosphorylated by the kinases ATR, ATM and DNA-PK in response to DSBs but also to stalled forks (Fig 1C). To quantify more precisely DSB, we used a direct marker of DSB, the phosphorylated form of 53BP1 at Ser1778. Indeed, phosphorylation of this site has been shown to correlate with the amount of DSB [15]. We found that 53BP1 is already significantly phosphorylated at mild dose of HU (Fig 1C) and accumulates in foci in replicating cells only 30 minutes after induction of DRS (S1B and S1C Fig). Importantly, because we extracted soluble proteins before cell fixation, we only quantified proteins on chromatin and therefore DSB bound p53BP1. A similar rapid accumulation of p53BP1 on chromatin of S-phase cells was found in response to treatment with aphidicolin, an inhibitor of the replicative DNA polymerases (S1D Fig). This phosphorylation of 53BP1 and its recruitment to chromatin was strongly inhibited by a chemical inhibitor of ATM (KU55933), indicating that it is dependent upon DSBs (Fig 1D). Together, these findings suggest that at least some stalled DNA replication forks are rapidly converted into DSBs during S phase in response to DRS, a phenomenon that we refer to as rapid replication fork breakage (RRFB).

### Rapid replication fork breakage occurs in a Mus81-independent manner

**Fig 2:**
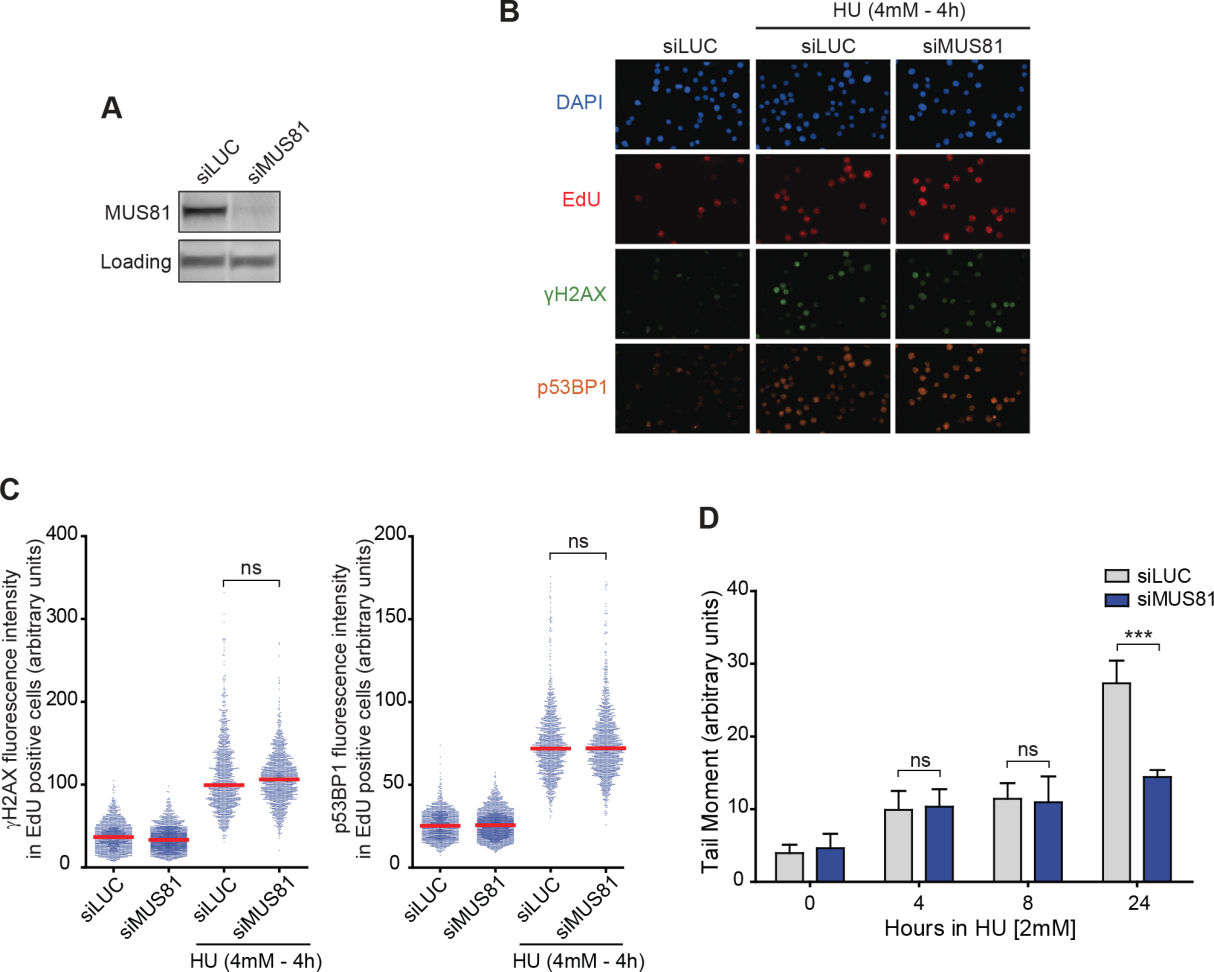
Rapid replication fork breakage (RRFB) is independent of MUS81. (A) Western blot of whole cell extracts of RKO cells showing depletion of Mus81 by siMus81 compared to a control, siLUC. Loading: nonspecific band. (B) Representative fields of QIBC images after MUS81 depletion and treatment with 4 mM HU for 4 hours. (C) Quantification of nuclear γH2AX (left) or p53BP1 (right) fluorescence intensity by QIBC in >1500 S-phase cells depleted of MUS81 (siMUS81) or depleted with a control siRNA (siLUC) and either treated or not treated with 4mM for 4 hours. ns: not significant (Mann–Whitney test). (D) Quantification of DSBs in cells depleted of MUS81 (siMUS81; blue) or depleted with a control siRNA (siLUC; gray) by neutral comet assay. Bars correspond to the median tail moment (% DNA in tail x tail length). Error bars represent standard deviations from 3 independent experiments in which at least 150 comets were scored. ns: not significant, ***p < 0.001 (Student’s T test).

It was previously reported that the formation of DSBs upon prolonged exposure (18–24 hours) to replication inhibitors requires MUS81 [5]. Thus, we addressed if its role in RRFB could have been overlooked for experimental sensitivity reasons. Despite strong depletion of MUS81 by RNA interference (Fig 2A), we saw no difference in the amount of γH2AX or p53BP1 in S-phase cells after DRS, as determined by QIBC (Fig 2B and 2C). To confirm this finding, we assayed the generation of DSBs upon HU treatment directly by neutral comet assay. Again, DSBs were detected at early time points both in cells depleted of MUS81 and in control cells (Fig 2D). After 24h of DRS, however, Mus81 depletion had a clear effect on the amount of DSBs, confirming previously published data [5]. Together, these data demonstrate that RRFB does not rely on the endonuclease MUS81.

### ARTEMIS and XPF-ERCC1 are involved in RRFB through two distinct pathways

**Fig 3:**
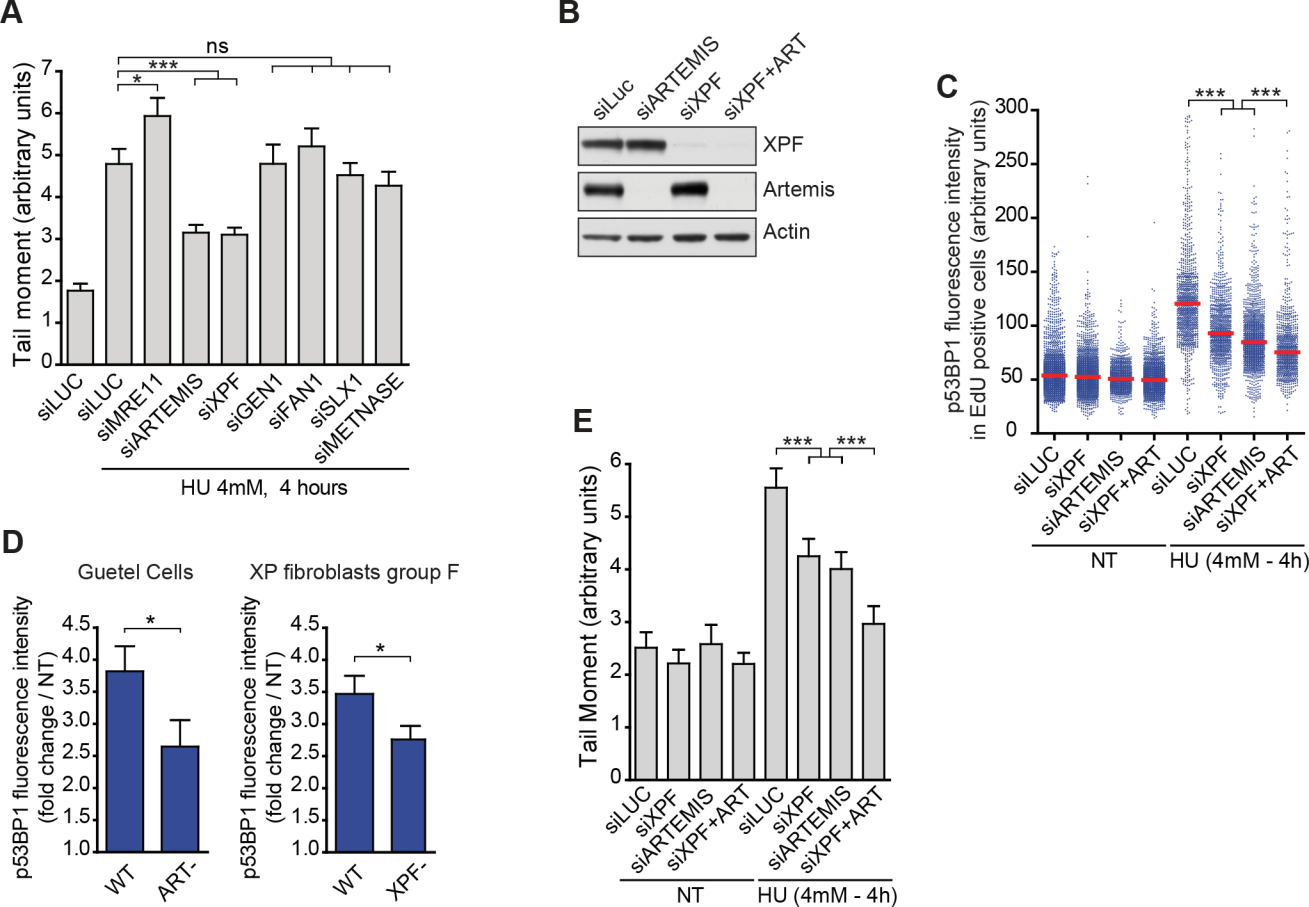
ARTEMIS and XPF are involved in RRFB. (A) Quantification by neutral comet assay of DSBs induced in response to treatment with 4 mM HU for 4 hours in control cells (siLUC) and in cells depleted of one of various endonucleases. The plot shows mean tail moment (% DNA in tail x tail length). Error bars represent SEMs. At least 150 comets were scored. *p < 0.05, ***p < 0.0001 (Mann–Whitney test). (B) Western blots of whole cell extracts of RKO cells showing depletion of Artemis (siARTEMIS), XPF (siXPF) and both (siXPF + ARTEMIS). (C) Quantification of nuclear p53BP1 fluorescence intensity by QIBC in >1500 S-phase RKO cells depleted of XPF and/or ARTEMIS and either untreated (NT) or treated with 4 mM of HU for 4 hours, as indicated. ***p < 0.0001 (Mann–Whitney test). (D) Quantification of nuclear p53BP1 fluorescence intensity by QIBC in Artemis-deficient RS-SCID patient cells (Guetel cells) and in XPF-deficient XP patient cells (XP fibroblasts group F) compared to WT primary fibroblasts. Mean fold change compared to untreated cells is shown. Error bars represent standard deviations from 3 independent experiments. *p < 0.05 (Student’s T test). (E) Quantification by neutral comet assay of DSBs in untreated RKO cells (NT) and in cells treated with 4 mM HU for 4 hours after depletion of XPF and/or ARTEMIS, as indicated. The plot shows mean tail moment (% DNA in tail x tail length). Error bars represent SEMs. At least 150 comets were scored. ***p < 0.0001 (Mann–Whitney test).

To investigate which other endonucleases might be involved in RRFB, we conducted neutral comet assay in RKO cells depleted of any one of several candidate endonucleases that might function during S phase (S2A Fig). Whereas depletion of most of them had no effect on the amount of DSBs, depletion of either ARTEMIS or XPF significantly inhibited DSB formation after 4 hours treatment with HU (Fig 3A). Similarly, in cells depleted of either Artemis or XPF (Fig 3B), we saw less p53BP1 on chromatin of S phase cells by QIBC when either ARTEMIS or XPF was depleted than we saw in control cells 4 hours (Fig 3C) but also only 1 hour after HU addition (S2B Fig) or after aphidicolin treatment (S2C Fig). Consistent with our findings with the depleted RKO cells, primary fibroblasts isolated from patients suffering from RS-SCID and deficient for ARTEMIS (Guetel cells) [16] or from a severe form of Xeroderma Pigmentosum and deficient for XPF [17] when treated with HU had less p53BP1 induction than had wild-type primary fibroblasts isolated from healthy donor (Fig 3D). Importantly, we found the same effects of ARTEMIS or XPF depletion in non-transformed RPE cells (S2D Fig). Interestingly, we observed cumulative effect on DSB and chromatin bound p53BP1 when both endonucleases were depleted (Fig 3C and 3E), strongly suggesting that ARTEMIS and XPF function independently to produce DSB under DRS.

**Fig 4:**
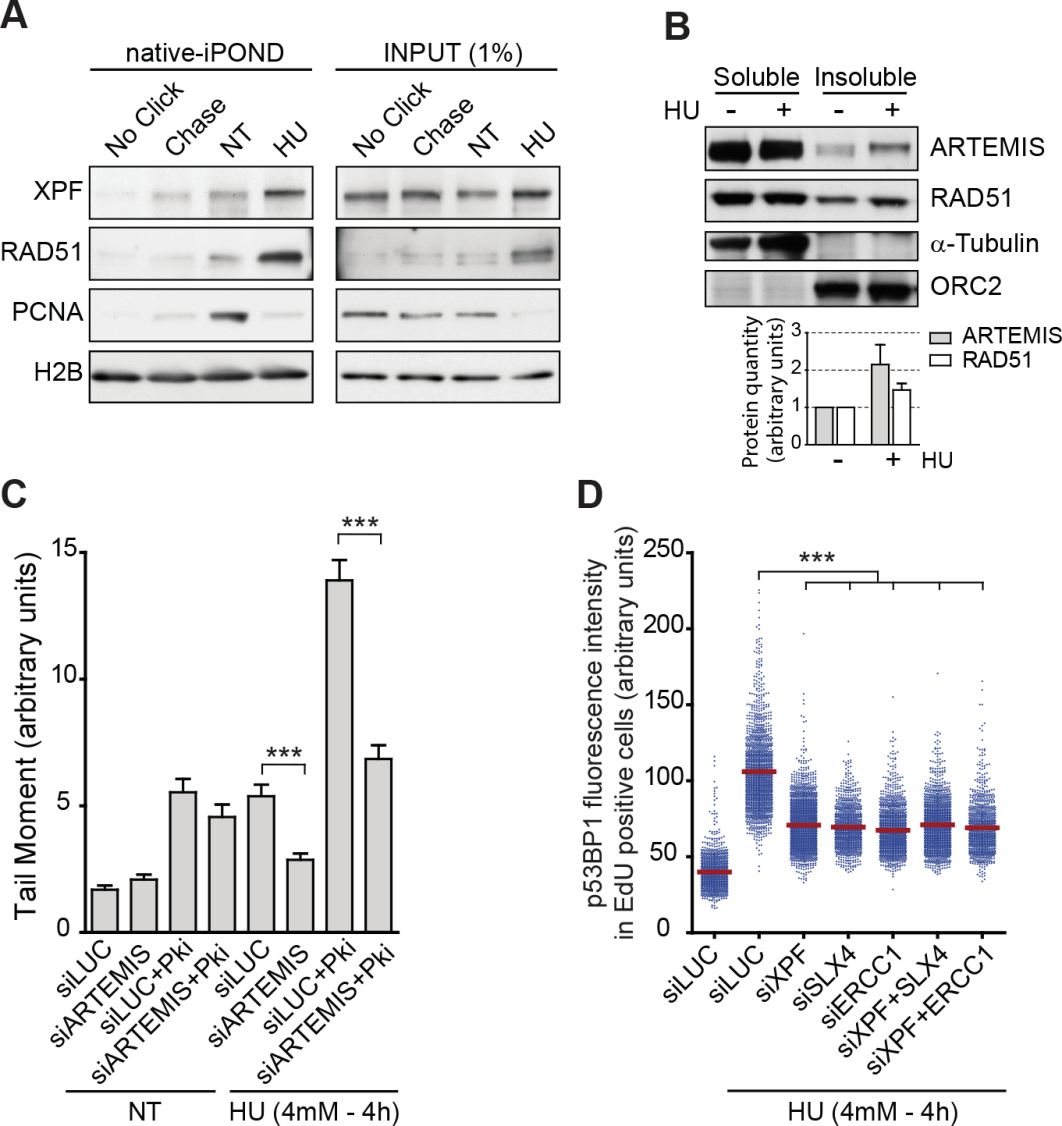
Regulation of ARTEMIS and XPF dependent RRFB. (A) Purification of fork-associated proteins by native-iPOND in response to HU (4mM– 4hours). No Click, negative control; Chase, chromatin behind the forks. (B) Western blots of the soluble and insoluble fractions of RKO cells either untreated (-) or treated with 4 mM HU for 4 hours (+). Bar graph shows Image-J quantifications of ARTEMIS and RAD51 bands normalized to ORC2 intensity. (C) Quantification by neutral comet assay of DSBs in control cells (siLUC) and in cells depleted of ARTEMIS (siARTEMIS) and treated or not for 4 hours with 4mM of HU and/or 2.5μM of DNA-PK inhibitor (NU7441). The plot shows mean tail moment (% DNA in tail x tail length). Error bars represent SEMs. At least 250 comets were scored. ***p < 0.0001 (Mann–Whitney test). (D) Quantification of nuclear p53BP1 fluorescence intensity by QIBC in >1500 S-phase RKO cells depleted of XPF, SLX4 and/or ERCC1 and treated with 4 mM of HU for 4 hours. ***p < 0.0001 (Mann–Whitney test).

To determine whether ARTEMIS and XPF act directly at stalled DNA replication forks, we performed iPOND, a methodology which allows the purification of fork-associated proteins. Here we used iPOND in native condition which has shown to improve capture efficiency and therefore sensitivity [18]. As previously reported [13], we observed less PCNA at replication forks of cells treated with HU compare to untreated cells, presumably because PCNA is unloaded from Okazaki fragments during HU treatment. Conversely more Rad51 was found at the stalled replication forks (Fig 4A). Moreover, we found more XPF in samples from cells treated with HU than in control cells, suggesting that XPF is recruited to stalled forks. We were unable to detect ARTEMIS by the native-iPOND method probably because the antibody was of too low affinity and iPOND captures very low amount of proteins even in native condition. However, we were able to detect it after fractionation of large amount of cells (Fig 4B). We found that ARTEMIS accumulates on chromatin (insoluble fraction) in the HU-treated cells, consistent with the idea that, like XPF, ARTEMIS may also be recruited to stalled DNA replication forks. Taken together, these data suggest that Artemis and XPF are recruited to stalled forks upon DRS to perform RRFB. It has been shown that DNA-PK activity is required for at least some ARTEMIS functions such as VDJ recombination [19]. Therefore, we tested by neutral comet assay if inhibition of DNA-PK could phenocopy ARTEMIS depletion after HU treatment. We found that cells treated with DNA-PK inhibitor accumulate DSB even in absence of replication stress (Fig 4C). Moreover, we observe a sharp induction of DSB upon DNA-PK inhibition and HU treatment. However, ARTEMIS depletion was still able to inhibit DSB formation in this condition. Thus, we concluded that ARTEMIS function in RRFB does not rely on DNA-PK activity. Moreover, XPF associates to ERCC1 to form an active holoenzyme and interacts with the scaffolding protein SLX4 which has been shown to regulate XPF-ERCC1 during replication stress [20]. Thus, we assessed the contribution of ERCC1 and SLX4 to XPF dependent RRFB by QIBC (Fig 4D). We found that ERCC1 or SLX4 depleted RKO cells exhibit less chromatin bound p53BP1 after HU treatment than control cells. Moreover, double depletion of XPF and ERCC1 or XPF and SLX4 did not yield to further reduction of chromatin bound p53BP1 compared to single siRNA transfected cells indicating that XPF, ERCC1 and SLX4 are all working in the same RRFB pathway.

### ARTEMIS—And XPF-ERCC1-dependent RRFB supports replication fork restart and prevents anaphase segregation defects

**Fig 5:**
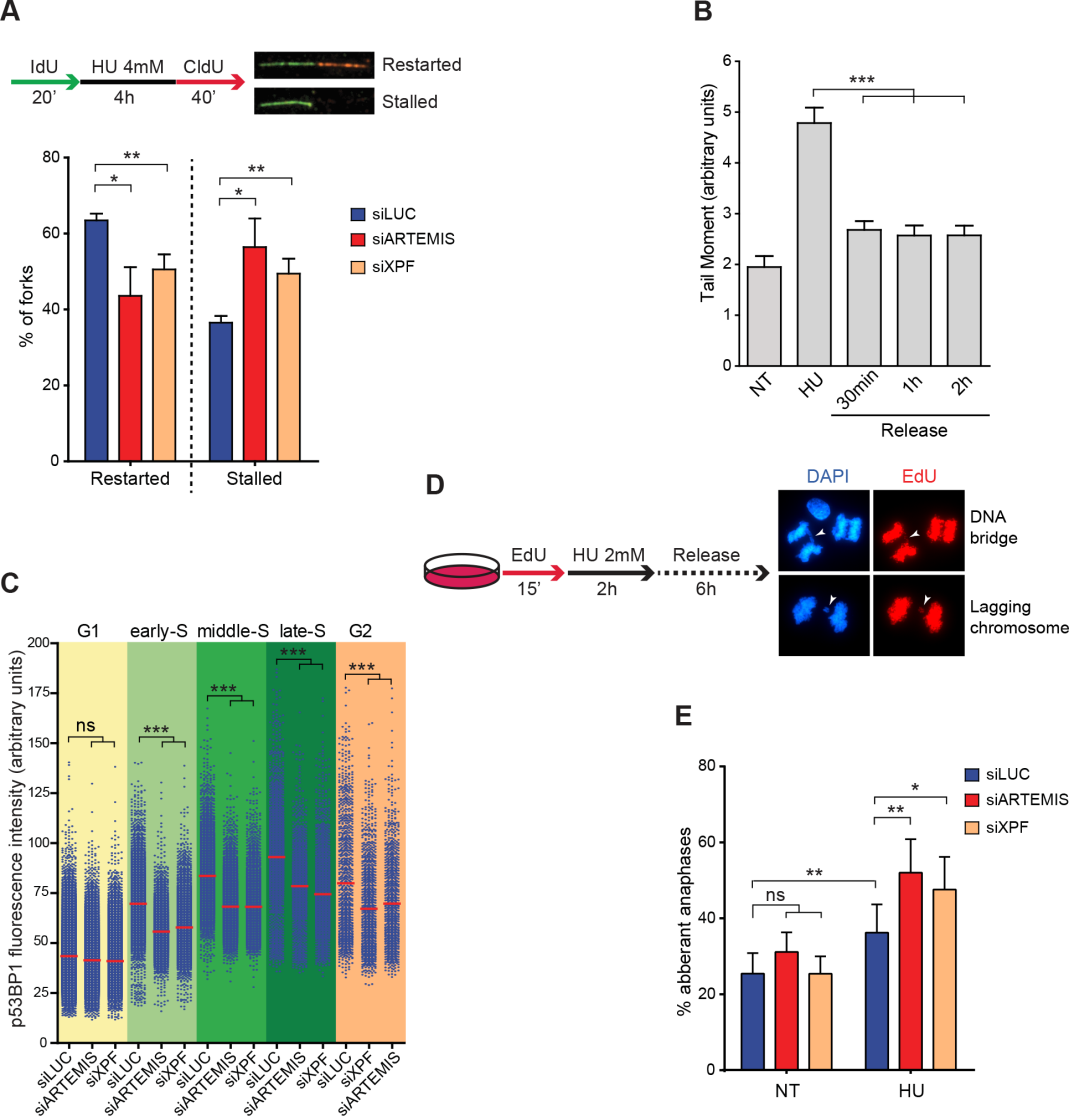
ARTEMIS and XPF promote efficient replication restart and prevent chromosome segregation defects. (A) Schematic representation of the DNA fiber assay with images of typical restarted or stalled forks (top). Means and standard deviations of the percentages of restarted or stalled forks were derived from three independent experiments. *p < 0.05, **p < 0.01 (Student’s T test). (B) RKO cells treated for 4 hours with 4mM HU and released into fresh media for the indicated times were subjected to comet assay to quantify DSBs. The plot shows mean tail moment (% DNA in tail x tail length). Error bars represent SEMs. At least 250 comets were scored. ***p < 0.0001 (Mann–Whitney test). (C) Quantification of nuclear p53BP1 fluorescence intensity by QIBC in >7500 cells depleted of Artemis (siARTEMISs) or XPF (siXPF) or treated with a control siRNA (siLUC) and treated with with 4mM HU for 4 hours. The cells were sorted into the five indicated phases of the cell cycle based on their EdU and DAPI content. ns: not significant, ***p < 0.0001 (Mann–Whitney test). (D) Schematic representation of the experiment performed to quantify aberrant anaphases in cells treated with HU and typical images of DNA bridges and lagging chromosomes in anaphase cells. (E) Quantification of aberrant anaphases in untreated (NT) or HU-treated cells depleted of ARTEMIS or XPF. Error bars represent standard deviations from 3 independent experiments. *p < 0.05, **p < 0.01 (Student’s T test).

To investigate whether RRFB induced by DRS affects replication resumption upon release from HU treatment, we used DNA fiber analysis to monitor fork restart. ARTEMIS—or XPF-depleted cells and control cells were incubated with IdU for 20 minutes to label ongoing replication forks and were subsequently exposed for 4 hours to 4 mM HU. The cells were then incubated without HU in medium containing CldU to label the restarted forks for 40 minutes, which was sufficient to observe restart of 65% of the forks in control cells (Fig 5A). In cells depleted of ARTEMIS or XPF, however, replication fork restart was significantly impaired and, conversely, the proportion of forks that remain stalled was higher than in the controls (Fig 5A). This observation suggests that some broken forks could be rapidly repaired to support fork restart. Therefore, we followed both by QIBC and comet assay the behavior of XPF and ARTEMIS dependent DSBs upon release from a HU block (Fig 5B and S3A Fig). Consistent with our replication restart data in control cells, we observed that most of the DRS induced DSBs were rapidly repaired within 30min. However, some DSBs remained unrepaired for at least 2 hours which might explain why not all the stalled forks are able to restart even in control cells, as observed by DNA combing.

Next we used QIBC to sort control and ARTEMIS—or XPF-depleted cells stained with EdU into early S phase, middle S phase, and late S phase according to their DNA content. Then, we analyzed the amount of p53BP1 on chromatin in the various S phase populations after HU treatment (Fig 5C). We observed increasing p53BP1 on chromatin from early to late S phase cells, suggesting that the number of broken forks increased as cells progressed through S phase. In cells depleted of ARTEMIS or XPF, we observed overall less p53BP1 at each phase than in the control cells, suggesting that ARTEMIS or XPF dependent fork cleavage is not restricted to early, mid or late S phase. Interestingly, we saw strong p53BP1 fluorescence intensity also in the G2 phase cells after HU treatment, showing that DRS induces DNA damage even when most of the genome is already duplicated. Moreover, the p53BP1 fluorescence intensity was significantly less in G2 phase cells when ARTEMIS or XPF were depleted (Fig 5C), suggesting that ARTEMIS—and XPF-dependent RRFB also occurs in G2 phase.

Although G2 phase cells have essentially completed DNA replication and so should synthesize relatively little DNA, RRFB occurs to a level comparable to early and middle S-phase. This observation suggests that DNA replication forks are more prone to breakage when cells approach mitosis. We hypothesized that efficient RRFB during G2 could support the completion of DNA replication before the cells enter mitosis and therefore prevent the accumulation of unresolved structures during chromosome segregation. To test this hypothesis, we treated EdU labeled RKO cells with 2mM HU for 2 hours, a dose and time that induces a significant number of broken forks (Fig 1A). We then removed the HU for 6 hours to allow S phase cells to reach mitosis where we quantified the proportion of lagging chromosomes and DNA bridges (henceforth referred as segregation defects) in EdU-positive cells (Fig 5D). We found that this short DRS resulted in a substantial increase of segregation defects in these cells. Importantly, prior depletion of either ARTEMIS or XPF from the cells aggravated the incidence of these defects (Fig 5E). To evaluate the incidence of RRFB on cell viability we treated RKO cells depleted of ARTEMIS or XPF for 4 hours with 4mM HU and assessed cellular viability by MTS assay 3 days later. Because HU could only impact the viability of cells that are in S-phase, we also evaluated their cell cycle distribution by FACS. Interestingly, while most of the replicating control cells were killed by the treatment, we found that ARTEMIS or XPF depletion confer resistance to HU (S3B Fig) Overall, these findings demonstrate that RRFB is important to support efficient DNA replication in S and G2 phase and to prevent chromosomal instability during mitosis. However if the amount of replication stress is too high RRFB could trigger cell death.

## Discussion

In this study, we describe a phenomenon in which DNA replication forks that stall in response to DRS are rapidly converted into DSBs, which we refer to as rapid replication fork breakage (RRFB). We identify two endonucleases, ARTEMIS and XPF-ERCC1 that promote RRFB at stalled replication forks during S and G2 phases in response to DRS to ensure efficient DNA replication and prevent mitotic segregation defects.

Interestingly, a previous study found that DSBs are induced poorly in Artemis-deficient cells by prolonged HU treatment [21]. This might be explained by the incapacity of these cells to cleave stalled replication forks early on. Similarly, another study found that depletion of the endonuclease scaffolding protein SLX4, which interacts with XPF-ERCC1, prevents the formation of replication-associated DSBs after long HU treatment (24 hours) [20]; the authors suggested that this function of SLX4 partially relies on XPF-ERCC1 but also SLX1 endonucleases. Here, earlier after DRS induction we clearly show that fork breakage is dependent on XPF-ERCC1 and SLX4 but not SLX1.

Why would the cell develop a mechanism of fork rescue that involve DSB is unclear. Our data demonstrate that RRFB is used to resume DNA replication at persistent stalled forks to prevent mitotic segregation defects. We show that RRFB also occurs at high frequency in G2 phase cells treated with HU. Thus, we speculate that RRFB might be used by the cell as the final attempt to rescue stalled forks. Indeed, several mechanisms have been described that help stalled forks resume DNA replication [22] and extensive fork remodeling such as fork reversal has been shown to occur upon DRS [23]. Interestingly, a recent study has shown that SAMHD1 promotes degradation of nascent DNA at stalled replication forks by stimulating the exonuclease activity of MRE11 which facilitates fork restart. Whether endo- and exo-nucleolytic activities work in concert at stalled replication forks need further attention. Importantly, the authors showed that exo-nucleolytic degradation of nascent DNA at stalled forks induces expression of pro-inflammatory type I interferons. As we observed that RRFB could trigger cell death when replication stress is excessive, both endo and exo-nucleolytic activities could represent new mechanisms to clear problematic cells which experience high replication stress from the tissues in order to prevent accumulation of mutations and therefore tumorigenesis.

How RRFB facilitates completion of DNA replication under DRS is still unclear. A recent study suggested that replication fork breakage might be managed by break-induced replication (BIR) during S phase in yeast [24]. This template switch-mediated repair might couple repair of the single-ended DSB induced by RRFB to completion of replication. It would also provide a rationale for the coexistence of Mus81 and RRFB nucleases. Indeed, MUS81 could resolve the D-loop induced by BIR in order to limit the mutagenic synthesis of POLD3 [24]. Recent evidence that BIR occurs in human cells and that Rad52 promotes replication restart after HU treatment also support this model [25, 26]. However, the potential importance of homologous recombination and end-joining mechanisms in the repair of broken forks cannot be ruled out.

Finally, because ARTEMIS and XPF-ERCC1 are structure-specific endonucleases, it is tempting to speculate that they might independently and specifically target several unusual DNA structures such as hairpins or cruciform structures at repeated sequences, R-loops at transcription sites or regressed forks. This hypothesis would explain the additive effects of depletion of ARTEMIS and XPF that we observed in our experiments. Interestingly, it has been shown that most of DSB arising from DNA replication stress are located within DNA repetitive sequences prone to form secondary structures such as hairpins but also at active transcription sites [27]. These genomic locations could be separately targeted by ARTEMIS and XPF-ERCC1 since ARTEMIS is known to cleave efficiently hairpins such as those present during VDJ recombination and XPF-ERCC1 has been shown to cleave DNA at active transcription sites that accumulate R-loops [28].

## Methods

### Cell lines

RKO cells were purchased from ATCC (CRL2577). Guetel cells derived from an ARTEMIS -deficient patient were originally obtained by the team of Jean-Pierre de Villartay (Paris, France) and fibroblasts derived from an XP patient of complementation group F (AS871 cells) were initially isolated by the team of Alain Sarasin (Villejuif, France) as well as the primary normal fibroblasts (AS405 cells). All cell lines used in this study were grown in DMEM medium (Gibco, Invitrogen, Paisley, UK) complemented with 10% fetal bovine serum (Lonza Verviers, Verviers, Belgium) and kept at 37°C in a humidified incubator containing 5% CO2.

### Transfection with siRNAs

The siRNAs used for this study were ON-TARGETPlus SMARTpool (Dharmacon). siRNAs were transfected to yield a final concentration of 50 nM. The cells to be transfected were seeded and then transfected by the ‘reverse transfection’ using the RNAiMax transfection reagent. Depletion of the target proteins was confirmed by western blot and by quantitative RT-PCR.

### Cell extracts and western blotting

Cellular fractionation was performed as described previously [29]. Whole cell extracts were prepared by lysing cells in buffer containing 40 mM Tris pH 7.5, 125 mM NaCl, 2mM MgCl2, 0.1% Triton X-100, 0.1 mM EDTA, 1 mM DTT, supplemented with protease inhibitors (Roche). The lysed cells were sonicated and boiled in Laemmli sample buffer for 5 minutes. Proteins were separated on NuPAGE Novex 3–8% Tris–acetate, or Bolt 4–12% Bis-Tris Plus gels (Invitrogen) according to the supplier’s instructions and then transferred onto PVDF blotting membrane (GE Healthcare) in FlashBlot transfer buffer (Advansta). The membranes were saturated by soaking for 1 hour in TBS containing 0.1% Tween and 5% non-fat milk. The antibodies and concentrations used are listed in S1 Table.

### Quantitative image-based cytometry (QIBC)

Cells growing on glass coverslips were incubated with 10 μM EdU for 15 minutes and then with hydroxyurea or aphidicolin (Sigma) at the concentrations and for the times indicated in the text. The soluble proteins were extracted by incubating in NuEx buffer (20 mM Hepes pH 7.4, 20 mM NaCl, 5 mM MgCl_2_, 0.5% NP40, protease-phosphatase inhibitors, 1 mM DTT) for 20 minutes and the cells were then fixed with 4% paraformaldehyde for 15 minutes at room temperature. The fixed cells were washed in PBS and the incorporated EdU was coupled to Alexa Fluor 647 by using the Click-iT EdU Alexa Fluor 647 Imaging Kit (Life Technologies) according to the manufacturer instructions. Subsequently, the coverslips were blocked with 5% BSA in PBS for 30 minutes and then incubated overnight at 4°C with primary antibodies (S1 Table) in PBS containing 1% BSA, 0.1% Tween 20. The coverslips were further washed with PBS and incubated with Alexa Fluor 488 or Alexa Fluor 555 secondary antibodies (diluted 1/1000) for 1 hour at room temperature. The DNA was stained with DAPI and the coverslips were mounted on microscopy slides in ProLong Diamond Antifade Mountant (Thermofisher). Images were taken with a Nikon Ni-E microscope and a DS-Qi2 camera equipped with a 20X objective. Fluorescence intensity was measured with the CellProfiler software (www.cellprofiler.org).

### Neutral comet assay

In Figs 1A, 3B and 4C, the neutral comet assays were performed with the CometAssay Kit (Trevigen) following the manufacturer’s instructions. In Fig 3A, the neutral comet assays were performed with a 4D Lifetank (4D Lifetec). Cells were mixed with low melting point agarose (0.5% final concentration) and then spotted onto 12-well gel spot plates (4D Lifetec). Samples were cooled to 4°C for about 10 min to solidify the gel. The cells were then lysed in 2.5 M NaCl, 0.1 M EDTA, 10 mM Tris (pH 10), 1% N-lauryl sarcosine, 0.5% Triton X-100, 10% DMSO for 1 hour at 4°C and then rinsed in electrophoresis buffer (100mM Tris pH 10, 150mM sodium acetate). The plates were incubated in electrophoresis buffer for 30 minutes and electrophoresis was performed at 1.1V/cm for 40 minutes at 8°C. The plates were then incubated with ethanol for 2 hours before drying. Comet DNA was stained with SYBRGold (Life Technologies) for 20 minutes, then rinsed with water and dried again prior to imaging with a Nikon Ni-E microscope and a DS-Qi2 camera equipped with a 10X objective. Comets were scored with CaspLab software (www.casplab.com).

### Isolation of proteins on nascent DNA in native conditions (native-iPOND)

Native iPOND was performed essentially as previously described [18] with the following modifications. Briefly, the Click-iT reaction was performed for 2 hours at 4°C. A Covaris S220 focused ultrasonicator was used for the sonication step at peak power 75 W, duty factor 20 and 200 Cycles/burst for 15 minutes at 6°C. Biotin-tagged nascent DNA was isolated by incubating the samples with Bio-AdemBeads Streptavidin Plus (Ademtech). Replication fork proteins were eluted by boiling in SDS sample buffer and analysed by western blotting.

### DNA fiber analysis

Cells were pulse-labelled with 50 μM ldU for 20 minutes. They were then incubated with 4 mM HU for 4 hours and followed by 40 minutes in medium containing 100 μM CIdU. The cells were harvested, lysed in 200 mM Tris-HCl pH 7.4, 0.5% SDS, 50 mM EDTA, and the DNA fibers were spread on glass slides. The slides were incubated with 0.5mg/ml pepsin in 30 mM HCl at 37°C for 20 minutes, the DNA was denatured in 2.5 M HCl for 1 hour and blocked with 1% BSA containing 0.1% Tween 20 in PBS. The nucleotide analogues were detected with primary antibodies against CldU (Novus), and IdU (BD Biosciences) and the secondary antibodies anti-rat Alexa Fluor 555 and anti-mouse Alexa Fluor 488 (Thermo Fischer Scientific). Coverslips were mounted on slides in Dako Fluorescence Mounting Medium (Dako). Images were captured with a Nikon Ni-E microscope and a DS-Qi2 camera equipped with a 20X objective and analyzed with NIS-Elements AR imaging software.

### MTS viability assay

Cells were reverse-transfected in triplicate in 96-well plates with control siRNA or siRNA targeting ARTEMIS or XPF. 48h later cells were treated with 4mM HU for 4 hours. Medium was changed and MTS was added 120 h after transfection of cells and incubated 2 h at 37°C. Viability was measured using a spectrophotometer at 590 nm.

### Quantitative RT-PCR analysis

After transfection with siRNAs, RKO cells were lysed and total RNAs were extracted using the RNeasy Mini Kit (Qiagen, Courtaboeuf, France), according to manufacturer's instructions. cDNAs were synthesized by using the Superscript II first-strand system for RT-PCR (Invitrogen, Milan, Italy). Quantitative real-time PCR was performed using the 7300 Real Time PCR System instrument with TaqMan (Applied biosystems) and using TaqMan probes indicated below (Applied Biosystems). Each sample was run in triplicate and was normalized to GAPDH expression.

TaqMan probes: ARTEMIS (Hs01052780_m1), FAN1 (Hs00429686_m1), GEN1 (Hs00416248_m1), SETMAR/METNASE (Hs01560192_m1), MRE11 (Hs00967443_m1), MUS81 (Hs01071851_g1), SLX1B (Hs02341353_g1) and XPF (Hs001193342_m1)

## Supporting information

S1 Fig Related to Fig 1. (A) Detection by western blot of DNA damage signaling from whole cell extracts of RKO cells treated with increasing doses of HU over 4 hours. (B) Representative QIBC images of RKO cells treated with 4mM HU over 2h showing p53BP1 staining in foci in EdU positive cells. (C) p53BP1 foci counting per nucleus obtained from QIBC experiments after treatment with 4mM HU over 2h. Each dot represent the mean number of foci for one experiment. Red bars represent the mean of three experiments. *p < 0.05, **p < 0.01, ***p < 0.001 (Student’s T test). (D) Quantification of nuclear p53BP1 fluorescence intensity by QIBC in > 2000 s-phase cells treated with increasing dose of Aphidicolin for 4 hours. ***p < 0.0001 (Mann-Whitney test).
(TIF)

S2 Fig Related to Fig 3. (A) qRT-PCR realized 24 hours after transfection of RKO cells. Gene expression has been normalized to GAPDH. (B) Quantification of nuclear p53BP1 fluorescence intensity by QIBC in >1500 S-phase RKO cells depleted of XPF or ARTEMIS and treated with 4 mM of HU for 1 hour. ***p < 0.0001 (Mann–Whitney test). (C) Quantification of nuclear p53BP1 fluorescence intensity by QIBC in >2000 S-phase RKO cells depleted of XPF or ARTEMIS and treated with 50μM of aphidicolin for 4 hour. ***p < 0.0001 (Mann–Whitney test). (D) Quantification of nuclear p53BP1 fluorescence intensity by QIBC in >1500 S-phase, normal and non-transformed RPE cells depleted of XPF or ARTEMIS and either untreated (NT) or treated with 4 mM HU for 4 hours, as indicated. ***p < 0.0001 (Mann–Whitney test).
(TIF)

S3 Fig Related to Fig 5. (A) Quantification of nuclear p53BP1 fluorescence intensity by QIBC in >1500 S-phase RKO cells treated with 4 mM of HU for 4 hour and released into fresh media for the indicated times. ***p < 0.0001 (Mann–Whitney test). (B) MTS viability assay of control cells (siLUC) or cells depleted of ARTEMIS or XPF and treated for 4 hours with 4mM of HU. In parallel cell cycle distribution has been assessed by FACS and the percentage of cells that are not in S-phase is graphed in red. Error bars represent standard deviations of three independent experiment. *p < 0.05 (Student’s T test).
(TIF)

S1 Table Antibody list and dilutions. (DOCX)
