# Supplementary figures and images for: DNA replication stress triggers rapid DNA replication fork breakage by Artemis and XPF
Source: PLoS Genet. 2018 Jul 30;14(7):e1007541. doi: 10.1371/journal.pgen.1007541 (PMC6085069; doi:10.1371/journal.pgen.1007541)

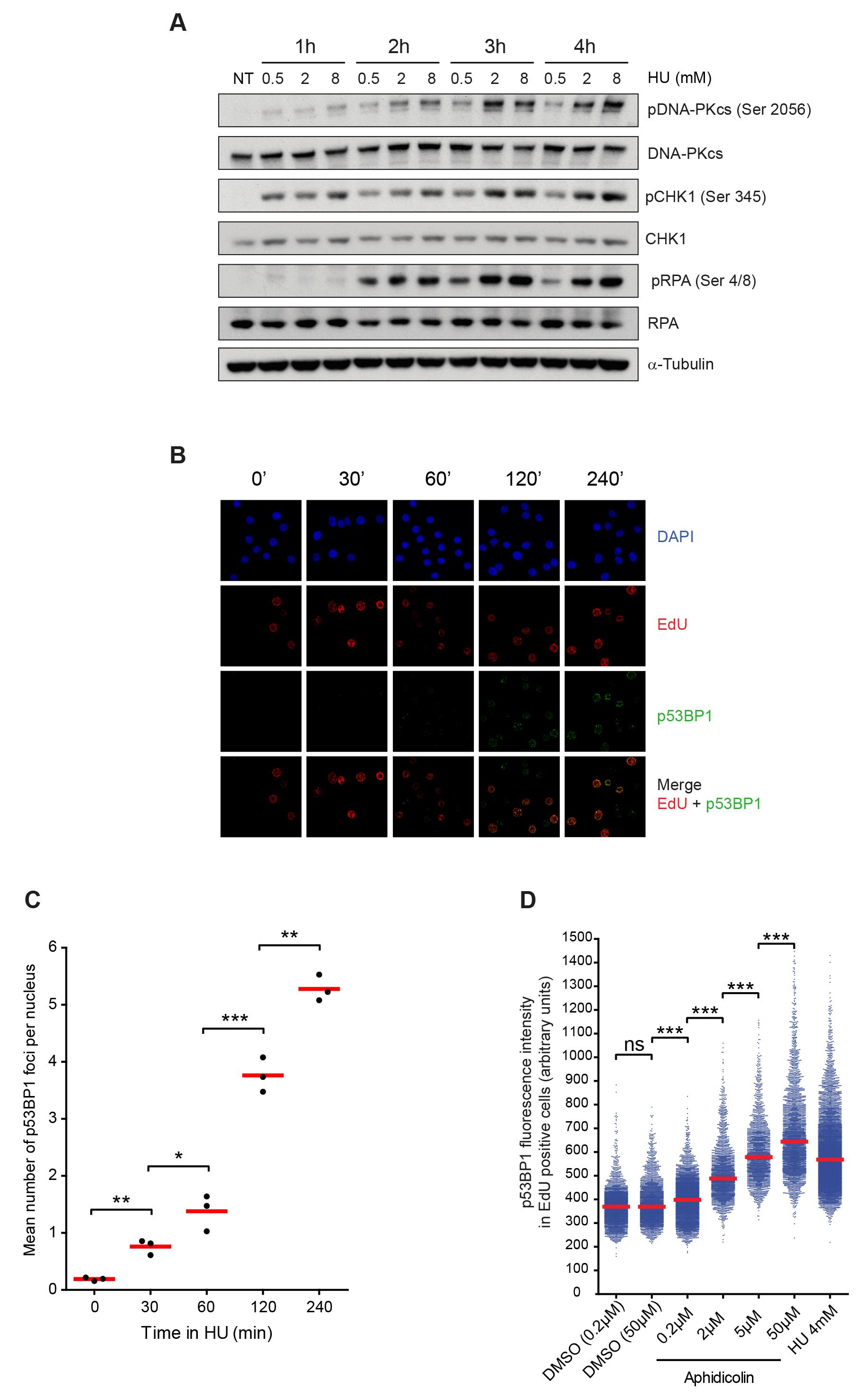

Supplement: S1 Fig — (A) Detection by western blot of DNA damage signaling from whole cell extracts of RKO cells treated with increasing doses of HU over 4 hours. (B) Representative QIBC images of RKO cells treated with 4mM HU over 2h showing p53BP1 staining in foci in EdU positive cells. (C) p53BP1 foci counting per nucleus obtained from QIBC experiments after treatment with 4mM HU over 2h. Each dot represent the mean number of foci for one experiment. Red bars represent the mean of three experiments. *p < 0.05, **p < 0.01, ***p < 0.001 (Student’s T test). (D) Quantification of nuclear p53BP1 fluorescence intensity by QIBC in > 2000 s-phase cells treated with increasing dose of Aphidicolin for 4 hours. ***p < 0.0001 (Mann-Whitney test). (TIF) [file pgen.1007541.s001.tif]

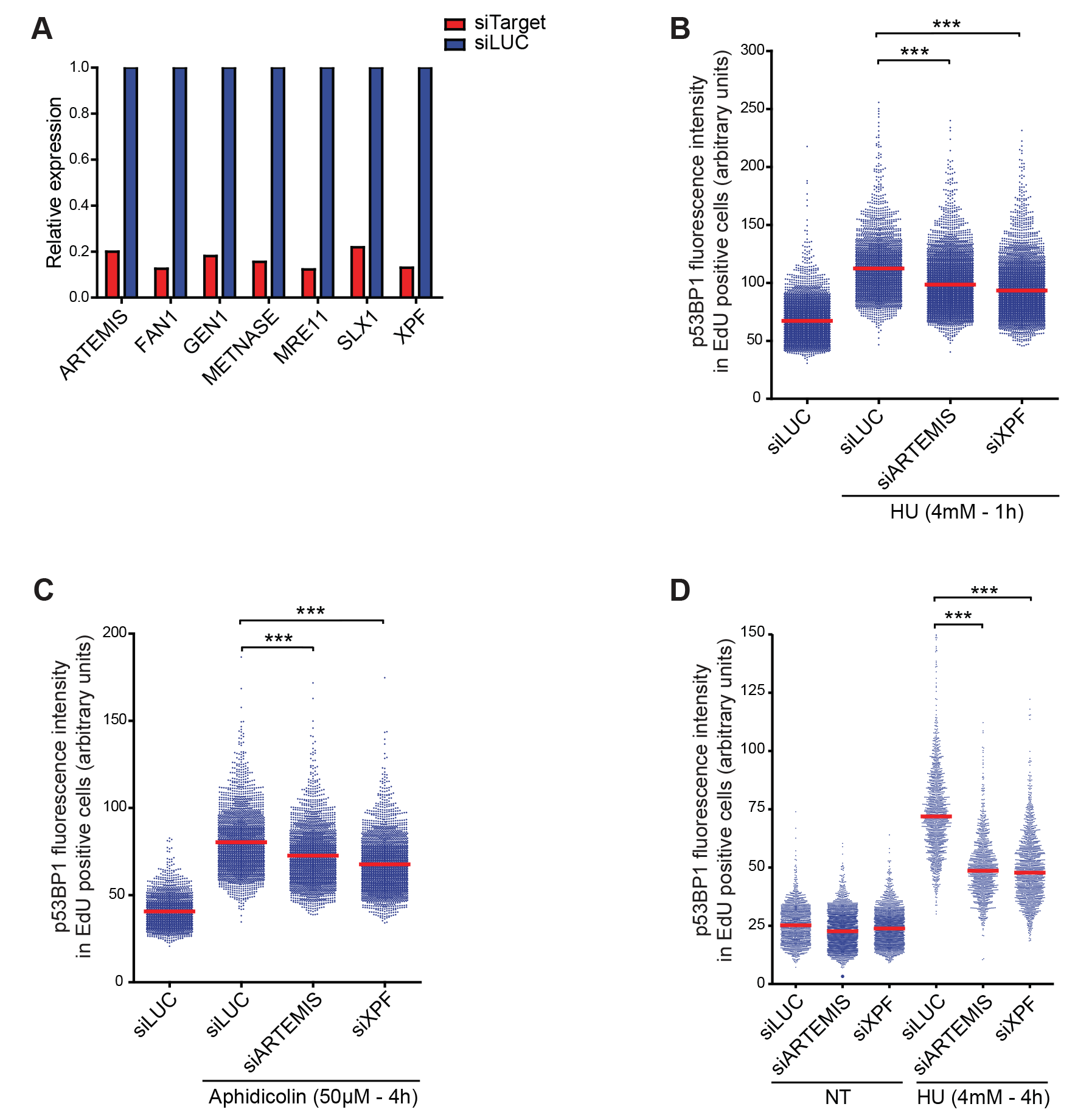

Supplement: S2 Fig — (A) qRT-PCR realized 24 hours after transfection of RKO cells. Gene expression has been normalized to GAPDH. (B) Quantification of nuclear p53BP1 fluorescence intensity by QIBC in >1500 S-phase RKO cells depleted of XPF or ARTEMIS and treated with 4 mM of HU for 1 hour. ***p < 0.0001 (Mann–Whitney test). (C) Quantification of nuclear p53BP1 fluorescence intensity by QIBC in >2000 S-phase RKO cells depleted of XPF or ARTEMIS and treated with 50μM of aphidicolin for 4 hour. ***p < 0.0001 (Mann–Whitney test). (D) Quantification of nuclear p53BP1 fluorescence intensity by QIBC in >1500 S-phase, normal and non-transformed RPE cells depleted of XPF or ARTEMIS and either untreated (NT) or treated with 4 mM HU for 4 hours, as indicated. ***p < 0.0001 (Mann–Whitney test). (TIF) [file pgen.1007541.s002.tif]

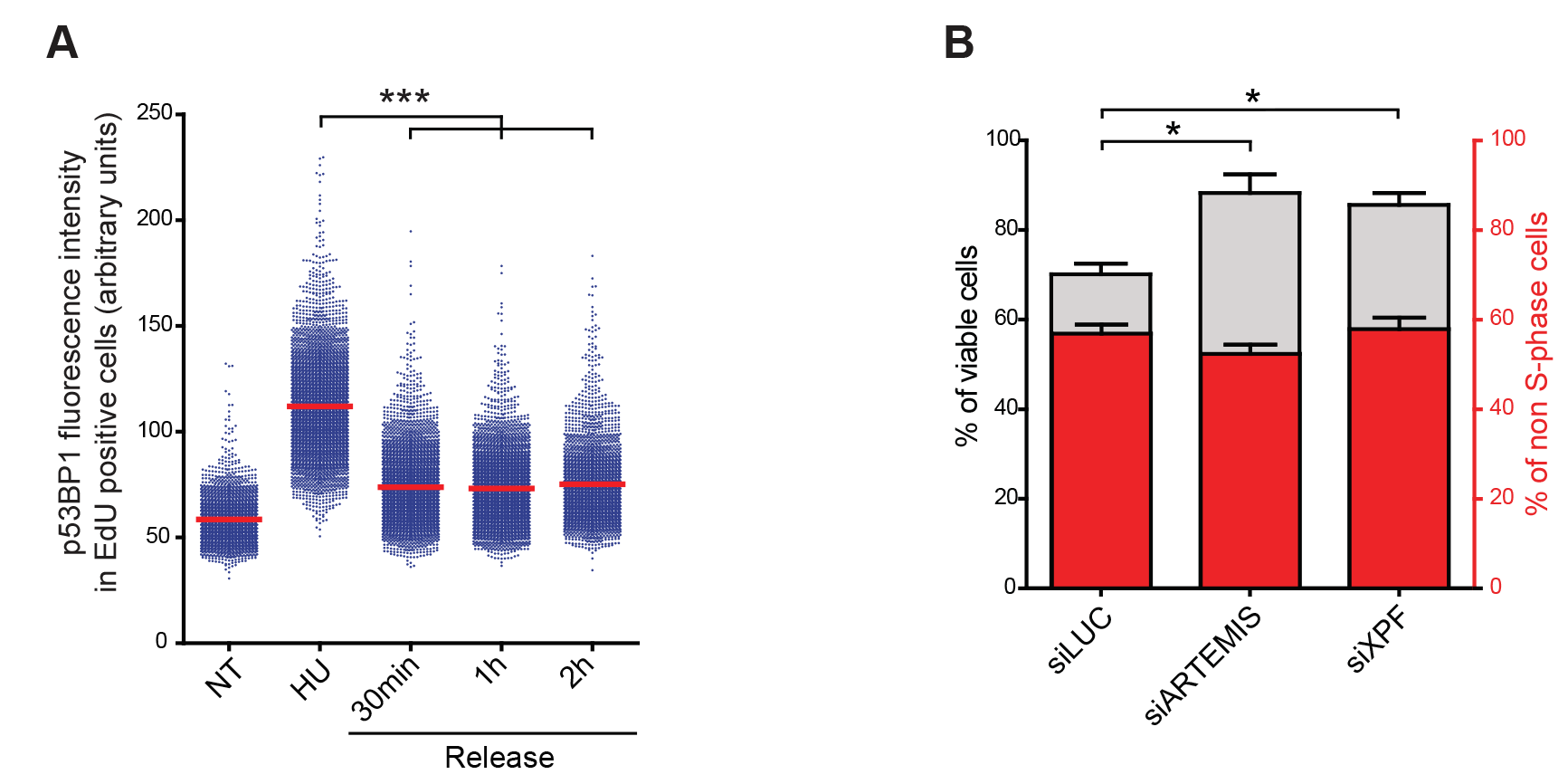

Supplement: S3 Fig — (A) Quantification of nuclear p53BP1 fluorescence intensity by QIBC in >1500 S-phase RKO cells treated with 4 mM of HU for 4 hour and released into fresh media for the indicated times. ***p < 0.0001 (Mann–Whitney test). (B) MTS viability assay of control cells (siLUC) or cells depleted of ARTEMIS or XPF and treated for 4 hours with 4mM of HU. In parallel cell cycle distribution has been assessed by FACS and the percentage of cells that are not in S-phase is graphed in red. Error bars represent standard deviations of three independent experiment. *p < 0.05 (Student’s T test). (TIF) [file pgen.1007541.s003.tif]
