# Supplementary material for: DNA replication stress triggers rapid DNA replication fork breakage by Artemis and XPF
Source: PLoS Genet. 2018 Jul 30;14(7):e1007541. doi: 10.1371/journal.pgen.1007541 (PMC6085069; doi:10.1371/journal.pgen.1007541)
Supplement: S1 Table — (DOCX) [file pgen.1007541.s004.docx]

Supplementary data

Table 1. Antibody list and dilutions.

| **Antibody** | **Source** | **Catalog Number** | **Diluition and use** |
| --- | --- | --- | --- |
| 53BP1 | Novus | 100-134 | 1/1000 (IF) |
| 53BP1 | Bethyl | 300-272A | 1/2500 (IF) |
| p53bp1 (S1778) | Cell Signaling Technology | 2675 | 1/200 (IF) |
| α-Actin | Sigma | A1978 | 1/50000 (WB) |
| Artemis | Cell Signaling Technology | 13381 | 1/500 (WB) |
| CHK1 | Santa Cruz | SC8408 | 1/1000 (WB) |
| pChk1 (S345) | Cell Signaling Technology | 2348 | 1/1000 (WB) |
| Cyclin A | Genetex | 73860 | 1/300 (IF) |
| DNA-PKcs | Abcam | ab1832 | 1/1000 (WB) |
| pDNA-PKcs (S2056) | Abcam | ab18192 | 1/1000 (WB) |
| H2B | Abcam | 1790 | 1/5000 (WB) |
| γH2AX(S139) cloneJBW301 | Millipore | 05-636 | 1/2500 (IF), 1/1000 (WB) |
| pH3 | Cell Signaling Technology | 9701 | 1/400 (IF) |
| Mus81 | Novus | NB100-2064 | 1/1000 (WB) |
| ORC2 | MBL | M055-3S | 1/1000 (WB) |
| PCNA | Santa Cruz | SC-56 | 1/1000 (WB) |
| RPA | Calbiochem | NA18 | 1/1000 (WB) |
| pRPA (S4 / S8) | Bethyl | A300-245A | 1/1000 (WB) |
| RAD51 | Santa Cruz | SC8349 | 1/500 (WB) |

IF – Immunofluorescence, WB - Western blot
